# Quantifying Training and Game Demands of a National Basketball Association Season

**DOI:** 10.3389/fpsyg.2021.793216

**Published:** 2021-12-21

**Authors:** Jennifer L. Russell, Blake D. McLean, Sean Stolp, Donnie Strack, Aaron J. Coutts

**Affiliations:** ^1^Faculty of Health, School of Sport, Exercise and Rehabilitation, University of Technology Sydney, Moore Park, NSW, Australia; ^2^Human and Player Performance, Oklahoma City Thunder Professional Basketball Club, Oklahoma City, OK, United States

**Keywords:** team sports, load monitoring, wearable technology, physical demands, NBA

## Abstract

**Purpose**: There are currently no data describing combined practice and game load demands throughout a National Basketball Association (NBA) season. The primary objective of this study was to integrate external load data garnered from all on-court activity throughout an NBA season, according to different activity and player characteristics.

**Methods**: Data from 14 professional male basketball players (mean ± SD; age, 27.3 ± 4.8 years; height, 201.0 ± 7.2 cm; body mass, 104.9 ± 10.6 kg) playing for the same club during the 2017–2018 NBA season were retrospectively analyzed. Game and training data were integrated to create a consolidated external load measure, which was termed *integrated load*. Players were categorized by years of NBA experience (1-2y, 3-5y, 6-9y, and 10 + y), position (frontcourt and backcourt), and playing rotation status (starter, rotation, and bench).

**Results**: Total weekly duration was significantly different (*p* < 0.001) between years of NBA playing experience, with *duration* highest in 3–5 year players, compared with 6–9 (*d* = 0.46) and 10+ (*d* = 0.78) year players. Starters experienced the highest *integrated load,* compared with bench (*d* = 0.77) players. There were no significant differences in *integrated load* or duration between positions.

**Conclusion**: This is the first study to describe the seasonal training loads of NBA players for an entire season and shows that a most training load is accumulated in non-game activities. This study highlights the need for integrated and unobtrusive training load monitoring, with engagement of all stakeholders to develop well-informed individualized training prescription to optimize preparation of NBA players.

## Introduction

In basketball, external training load data can inform decision-making regarding periodization (Schelling and Torres-Ronda, 2013) and injury reduction strategies (Caparrós et al., 2018), which may lead to optimized player health and physical performance (Halson, 2014). External “training load” is a construct encompassing the training stimulus imposed on players by both practices and competitions, and its quantification can be achieved using various proxy measures, such as distance or accelerometer load (Impellizzeri et al., 2019). In team sports, such as basketball, where different modes of training are often completed, practitioners may be required to use several different measures to quantify the overall training load in practice and competition (Buchheit et al., 2014). For instance, players in the National Basketball Association (NBA) may wear technology/devices during practices that are not permitted during games (McLean et al., 2018), while optical tracking (OT) technology (Second Spectrum Los Angeles, United States) is used during games to quantify external load, but these systems are not available in practice settings.

While a variety of technologies (e.g., wearables and OT in basketball) may report similar load metrics, limited understanding about agreeability of these systems can lead to issues when combining data, particularly considering how raw data is collected and analyzed, which may affect the final metrics. Because the relationship among multiple systems is poorly understood, much of the current basketball literature reports external load data isolated to either practice or competition (Teramoto et al., 2017; Caparrós et al., 2018; Lewis, 2018), with limited research describing integrated, season-long load demands. For NBA players specifically, current research on the external load demands has been limited to competition only (Caparrós et al., 2018; Lewis, 2018). Over a 6-month regular season, NBA teams play 82 games at an average frequency of 3.4 games/week (McLean et al., 2018), which is considerably higher than other professional basketball leagues. As only half of the days in the regular season include games, there is a significant amount of time available for non-game court work or recovery. Despite this significant amount of time available for non-game activity, there is currently no study that describes the combined practice and game load demands in professional basketball throughout an entire season.

Describing external load based on individual player characteristics is also important in better understanding the training dose-response relationship over time. Differences in basketball player characteristics that have been previously investigated include position (Svilar et al., 2018; Salazar et al., 2020a) and playing rotation status (Conte et al., 2018; Vazquez-Guerrero et al., 2018). Professional basketball can employ a wide range of experience levels, from draftees right out of college to veteran players who have been in the NBA for decades. The differences in age and years of experience may affect loading demands and therefore impact preparation strategies. However, these characteristics have never been examined and reported in the NBA across an entire season.

Quantification of the holistic on-court demands is needed to better understand training stimuli in professional basketball players. Therefore, the primary objective of this study was to integrate external load data garnered from practices and games to describe the physical demands of an NBA season. Additionally, this study described the seasonal training load according to player’s playing position, years in the league, and game rotation status.

## Materials and Methods

### Participants

Data from 14 professional male basketball players (mean ± SD; age, 27.3 ± 4.8 years; height, 201.0 ± 7.2 cm; body mass, 104.9 ± 10.6 kg) from the same NBA club were retrospectively analyzed for this study. Data were included for players who were under contract with the same club for the entire regular season and excluded if they had a two-way contract (e.g., player is contracted to play for both NBA team and its developmental team affiliate). The study was approved by the Human Research Ethics Committee of the University of Technology Sydney (UTS; HREC # ETH18-2658), and consent was granted by the NBA and the NBA Players Association as per the guidelines and requirements for “NBA related health research” governed by the NBA Collective Bargaining Agreement (CBA; NBA.com, 2017).

### Experimental Design

A longitudinal, observational design was employed for this study. External training load and duration data were collected during the 2017–2018 NBA season (September to April), which included 3 weeks of pre-season and 26 weeks of the regular season. Post-season data were excluded from this analysis.

### Methodology

All practices and games were included and assigned to the following activity categories: team training (basketball-specific court work done as a team), official NBA games (“games”), and individual training (basketball-specific court work not done with the team). Court work was further categorized by drill type, including (1) Skill drills, which are predominantly scripted drills with limited physical contact, focused on skill development, (2) Simulated play, which are predominantly non-scripted drills, focused on game-like physical contact, pace, and situations, and (3) official NBA game play. Court work was also characterized by tactical emphasis of the drill (offensive, defensive, both), and players were characterized by playing position, years of NBA experience, and playing rotation (Table 1).

**Table 1:** Categorization of activities and participants.

	Category	Definition
**Activity categories**		
Drill Type	Skill^*^	Predominantly scripted drills with limited physical contact, focused on skill development or team tactics.
	Simulated play^**^	Predominantly non-scripted drills, focused on game-like physical contact, pace, and situations.
	Game play^***^	Any league mandated competitive event.
Tactical Emphasis	Offensive	Predominantly offensive emphasis and physical demands.
	Defensive	Predominantly defensive emphasis and physical demands.
	Both	Approximately equal offensive and defensive strategy and physical demands.
**Participant categories**		
Playing Position	Backcourt	Point guards; Shooting guards
	Frontcourt	Small forwards; Power forwards; Centers
Playing rotation	Starter	Started ≥90% of games played with mean of ≥25 min per game.
	Rotation	Played in ≥70% of regular season games with mean of 13–22 min per game.
	Non-Rotation	Mean of <5 min per game over the course of the season.
Years in NBA	1–2	Defines number of years active on an NBA or NBA G-League affiliate roster.
	3–5	
	6–9	
	10 +	

* *Entire activity duration collected for all players participating at any point in activity*;

** *Activity duration collected only during “live” parts of the drill (i.e., excluding breaks), for only the participants actively in the drill;*

*** *Activity duration collected only when game clock is running and during inbounds plays after referee hands basketball to player to inbound*.

External training load data from an ultrawideband (UWB) local positioning system (Catapult ClearSky, Catapult Sports, Melbourne, Australia) and inertial measurement unit (Catapult T6, Catapult sports, Melbourne, Australia) were integrated with external game day load data from an OT system (Second Spectrum, Los Angeles, United States) to quantify external load across all on-court activities. The process of merging external load data from two different measurement systems has been evaluated in professional soccer (Buchheit et al., 2014; Taberner et al., 2020; Ellens et al., 2021), with findings that suggest positional data can be interchanged between different systems to confidently quantify external load (Buchheit et al., 2014; Taberner et al., 2020; Ellens et al., 2021). The two systems used for load quantification in this study were evaluated simultaneously during basketball-specific activity (e.g., running, change of direction, and 5 on 5 basketball play) to determine the level of agreement, *via* regression analysis, of external load metrics. On an individual player basis, regression equations were generated between the OT system and UWB system. These equations indicated strong relationships for each subject (R^2^ ranging from 0.93 to 0.99) for total distance and PlayerLoad^™^ (PL). The resulting regression equations were used to convert OT distance from NBA games to an equivalent PL metric. While this type of load quantification in team sports with similar technology has a level of error associated with merging data, the error is not expected to outweigh the practical implications of weekly load monitoring (Taberner et al., 2020). Indeed, this novel approach to integrating external load demands from NBA practices and games is the only approach that allows for a consistent external load measure throughout an entire season based on league restrictions around load monitoring (NBA, 2017; McLean et al., 2018).

During training sessions, external load data were collected by players wearing a microsensor device (Catapult T6, Catapult Sports, Melbourne, Australia) voluntarily, as per NBA CBA stipulations (NBA.com, 2017). The microsensor was worn in a tight-fitting manufacturer-provided garment, positioned between the scapulae according to manufacturer specifications, sampling inertial data at 100 Hz. Throughout the season, 10 players elected to wear the device, and participation in non-game court activities was recorded using microsensor manufacturer software (Catapult Openfield, Version 1.18, Catapult Sports, Melbourne, Australia). Data collected from court work were included in *integrated load* analyses if players wore their device for at least 95% of the non-game court work sessions (except for pre-game court work). Pre-game court work remained relatively consistent for each player across all 82 regular season games; therefore, small samples taken of each player’s pre-game session were used to estimate individual pre-game training load. For situations in which microsensor data were not collected during non-game court work for the players that elected to wear the unit (e.g., system errors, unit malfunction, and pre-game), *integrated load* was estimated per drill on an individual basis, using measured duration and historical load per minute values from each player for similar drill categories (Bowen et al., 2017). As a result, approximately 18.8% of the *integrated load* data used in this study was estimated (1857 ± 422 min).

If a player did not wear the microsensor unit regularly (*n* = 4), a device was assigned to that player, and their non-game court activities were recorded with the same methods outlined above. The resulting data were included in duration analyses only. After all training sessions, wearable data were downloaded using the manufacturer software package, then exported to Microsoft Excel (Microsoft Office, 2016, Washington, United States) for integration. Game data were collected *via* the NBA contracted OT system (Second Spectrum, Los Angeles, United States), sampling at a rate of 25 frames per second. The data were then processed and exported in a JavaScript Object Notation file by the company which manages the optical tracking cameras (Second Spectrum, Los Angeles, United States), converted to Comma-Separated Value format using a customized script in R (R Foundation for Statistical Computing, Vienna, Austria) and imported locally to Microsoft Excel for integration. Once these data streams were integrated, all data were exported to SPSS (IBM SPSS Statistics for Macintosh, Version 26.0., IBM Corp., New York, United States) for analysis.

### Statistical Analysis

Dependent variables (*integrated load*, duration) were summed in weekly blocks from Monday to Sunday per player and described descriptively (mean, SD, 95% confidence intervals (CI)). While the NBA game schedule does not follow a consistent weekly schedule, planning and periodization of court work for this team was conducted on a weekly basis following a Monday-Sunday block. From the 29 available weeks, 14 players duration data (*n* = 406 player weeks) and 10 players *integrated load* data (*n* = 290 player weeks) was included in the final analyses. Data were included in weekly sum analyses if the daily collection time was greater than 30 s. Differences in dependent variables based on activity categories, drill types, and tactical emphasis were also analyzed descriptively. Mixed models were used to compare means of total weekly integrated load and duration, between playing position, years in the league, and game rotation status. Players were treated as a random effect with scaled identity covariance matrix. Mixed models were used for their ability to model possible correlations of residual errors within each player over time. Model’s residuals were visually inspected for normality and outliers (± 3 SD) and the predictors estimated marginal means were compared between groups with a Bonferroni correction.

Effect sizes were calculated to assess practical significance of differences and were considered: ≤0.2, trivial; >0.2–0.6, small; >0.6–1.2, moderate; >1.2–2.0, large; 2.0–4.0, very large (Hopkins et al., 2009). Years playing in the NBA were binned together (seasons 1–2, seasons 3–5, seasons 6–9, and seasons 10+). Only one participant who agreed to wear the microsensor device played in the NBA for 10+ and was excluded from the years in the NBA analysis due to insufficient sample size. All statistical analyses were performed in SPSS Version 22.

## Results

**Figure 1:**
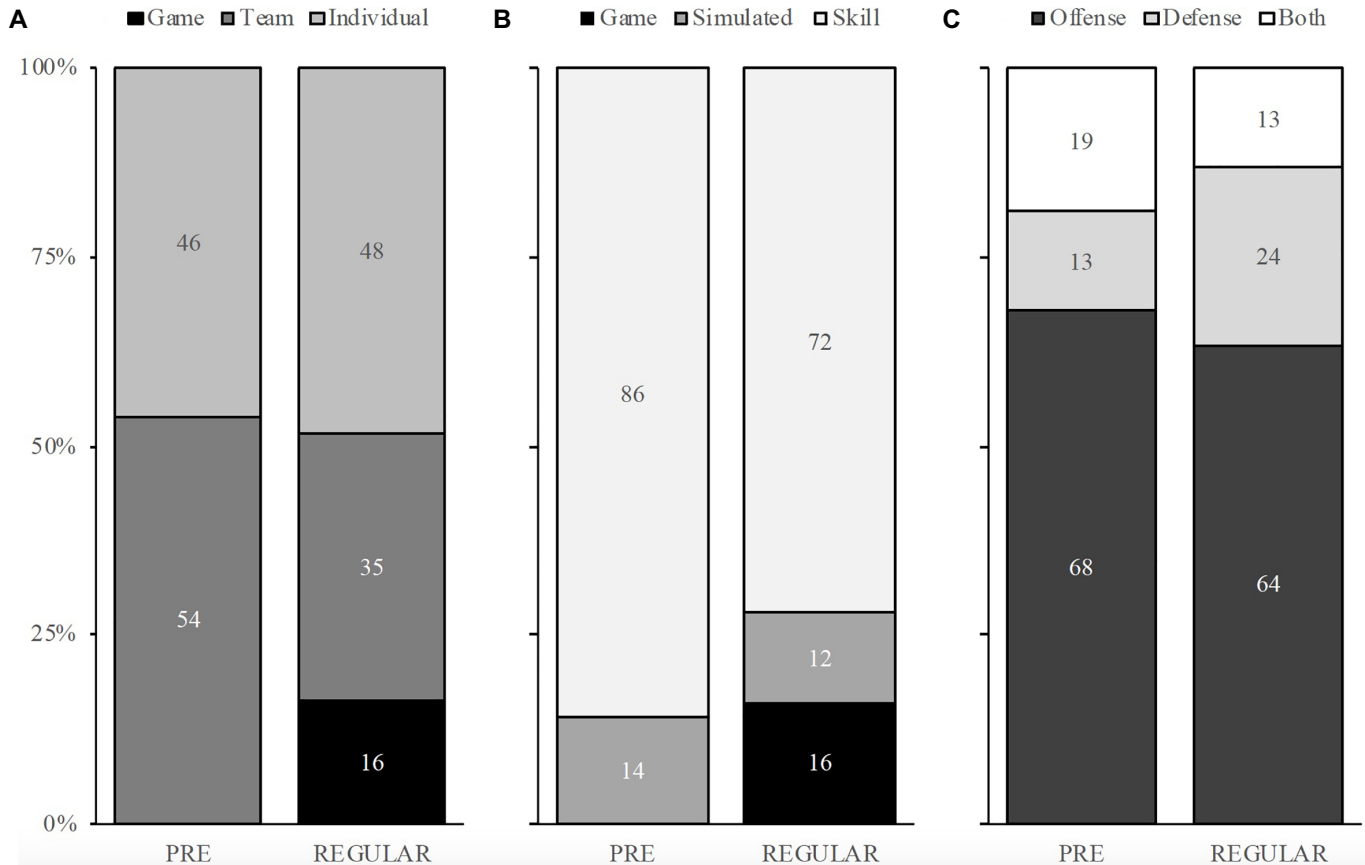
Percentage duration spent on-court work in pre-season (PRE) and regular season (REGULAR) based on activity category **(A)**, drill type **(B)**, and tactical emphasis **(C)**. Game = any league competitive event; Team = basketball-specific court work done as a team, Individual = basketball-specific court work not done with the team, Simulated = predominantly non-scripted drills, focused on game-like physical contact, pace, and situations, Skill = predominantly scripted drills with limited physical contact, focused on skill development, Offense = basketball activity with predominantly offensive emphasis, Defense = basketball activity with predominantly defensive emphasis, Both = basketball activity with equal offensive and defensive emphasis.

**Figure 2:**
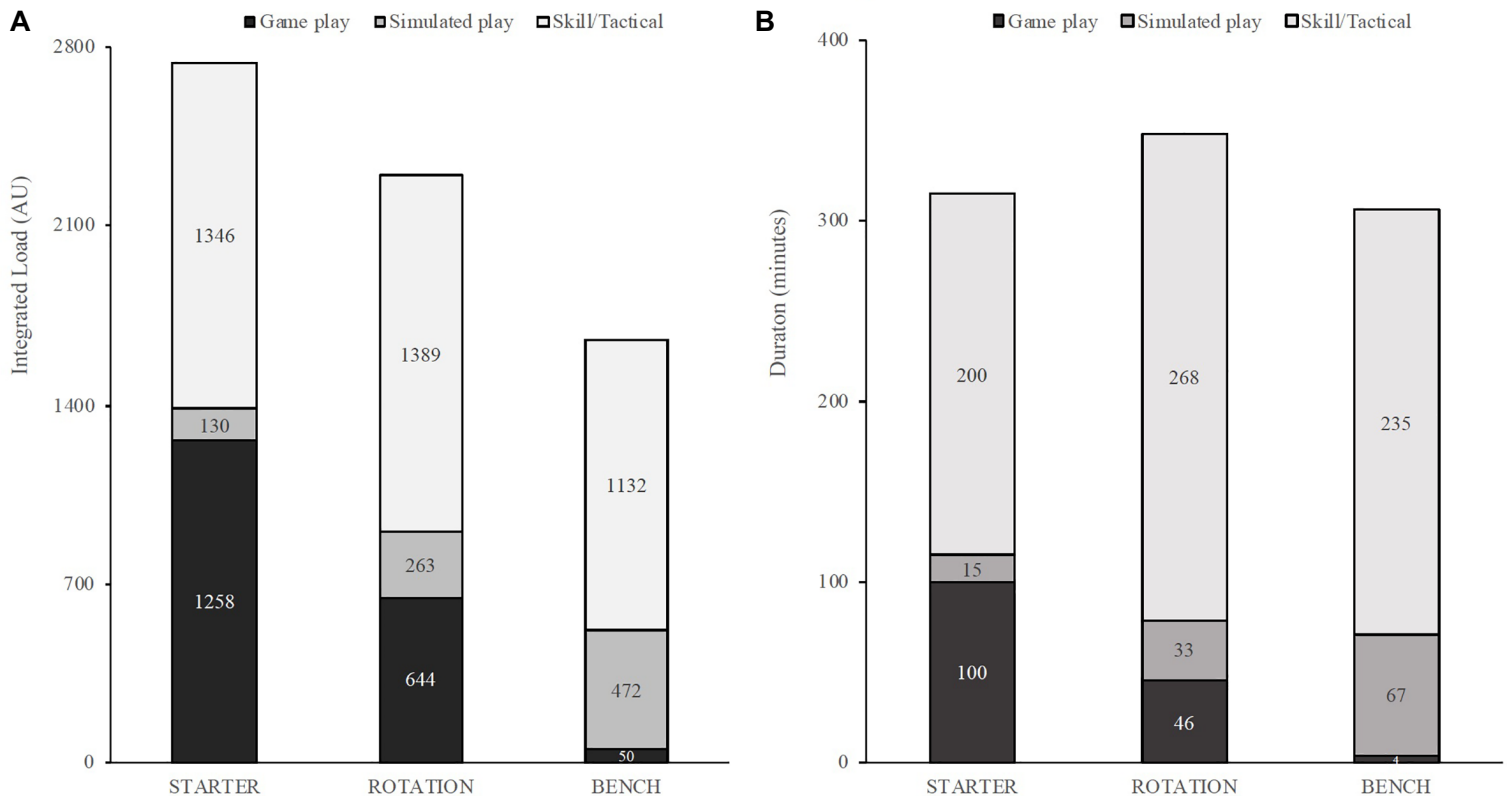
Average weekly *integrated load* **(A)** and duration **(B)** during the regular season, by rotation status.

Descriptions of mean weekly duration spent in different activity categories, drill types, and tactical emphasis are shown in Figure 1 based on seasonal phase and shown in Figure 2 based on player rotation status, respectively.

Mixed Models showed significant effects for rotation status on *integrated load* (*F*(2, 7.41) = 15.19, *p* = 0.002). Post-hoc comparisons showed bench players had notably lower integrated load than starters (*p* = 0.003, *d* = 0.77) and rotation players (*p* = 0.013, *d* = 0.46). Comparisons between starters and rotation players were insignificant (*p* = 0.20, *d* = 0.28). Models showed insignificant effects for years in NBA (*F*(2, 6.01) = 2.25, *p* = 0.19) and position (*F*(1, 7.92) = 0.02, *p* = 0.89) on *integrated load*.

Mixed models showed significant effects for years in the NBA on duration (*F*(3, 95.14) = 19.06, *p* < 0.001). Post-hoc comparisons showed that those played 10+ years had significantly lower duration than those that played 6–8 years (*p* = 0.043, *d* = 0.32), 3–5 years (*p* < 0.001, *d* = 0.78) and those that played between 1 and 2 years (*p* < 0.001, *d* = 0.61). Those that played 6–8 years had significantly lower duration than those who played 3–5 years (*p* = 0.001, *d* = 0.46). Models showed insignificant effects for Rotation status (*F*(2, 10.91) = 0.70, *p* = 0.52) and position (*F*(1, 11.95) = 0.42, *p* = 0.53) on duration.

Estimated marginal means, standard deviations and 95% confidence intervals can be seen in Table 2.

**Table 2:** Descriptive statistics of total player weeks of *integrated load* and duration across participant categories.

	*Integrated Load* (AU)				Duration (minutes)				n	EMMean	95% CI	ES, Interpretation	n	EMMean	95% CI	ES, Interpretation
**Total**	278^1^	2,192	1899, 2,485		394^2^	340	314, 365	
Frontcourt	220	2182.548	1829, 2,536	0.01, Trivial	278	345	314, 376	0.06, Trivial
Backcourt	58	2230.396	1,524, 2,937		116	327	278, 377	
1–2 years^A^	87	2134.934	1708, 2,562	0.18, Trivial^A-B^	86	365	342, 388	0.17, Trivial^A-B^
3–5 years^B^	104	2504.795	2,135, 2,875	0.06, Trivial^B-C^	105	388	367, 409	0.46, Small^B-C^
6–9 years^C^	58	2006.12	1,483, 2,529	0.26, Small^C-A^	87	327	304, 350	0.32, Small^C-D^
10+ years^D^	–	–	–	–	116	284	264, 304	0.61, Moderate^D-A^
								0.78, Moderate^D-B^
								0.29, Small^C-A^
Starter^E^	46	2664.792	2,328, 3,002	0.28, SmallE-F	134	329	285, 374	0.09, Trivial^E-F^
Rotation^F^	145	2302.679	2092, 2,513	0.46, SmallF-G	174	356	316, 397	0.1, Trivial ^F-G^
Bench^G^	87	1699.125	1,427, 1971	0.77, Moderate^E-G^	86	324	267, 382	0.01, Trivial^E-G^

*EMMean, Estimated Marginal Mean; ES, Cohen’s D effect size (* Hopkins et al., *2009* *); n, number of player weeks; CI, confidence interval; AU, arbitrary units. 1,2: Number of outliers removed.*

## Discussion

This study described the weekly load and duration demands for NBA players throughout an entire season (i.e., game and practice). NBA players spend a large proportion of time in non-game court work (84% total duration on court), and training load is highest for starters and players with 3–5 years of NBA experience. There were no meaningful differences in training load or duration between different positional groups. This study provides a novel model for integrating load data from practices and games in the NBA. The differences in training requirements between groups according to rotation status highlight the importance of holistic, unobtrusive training load monitoring in the NBA.

This study described the time spent in different on-court training activities across an entire NBA season. Given the congestion of the NBA playing schedule (Esteves et al., 2020; Yang et al., 2021), it is interesting that most active time on-court is spent in non-game activities. Load management strategies in the NBA commonly include reducing game exposure (Scamardella et al., 2020), and the findings of this study suggest that there is ample opportunity to manage exposure during non-game court activity to reduce external load demands over the course of a season. While games themselves regularly have 48 min of clock time when players are actively engaged in basketball, this activity actually takes place over a 2–3 h period. This distinction between “active” time and “total” time is especially important in basketball, where duration quantification methods are often poorly described (Russell et al., 2021). Additionally, while this study was the first to report load and duration measures across an entire NBA season, the rate of load accumulation (i.e., training intensity) was not described, which should be investigated in future studies.

Our findings reveal trivial differences between total time on-court based on playing rotation status, but show *integrated load* was notably higher for starters and rotation players compared to bench players (*d* = 0.77; *d* = 0.46). Further starters had visually higher *integrated load* than rotation players (*d* = 0.28), but this may have occurred by chance. These differences between playing rotation status are interesting given the similar amount of time spent on-court in basketball-related activities (starters = 329 min/week, rotation = 356 min/week, bench = 324 min/week), with meaningful differences evident between the type of activities completed. Logical findings were that starters had more playing time in games than both rotation and bench players (see Figure 2B) while bench players spent the most time in simulated play drills, most likely due to attempts to replicate the demands of the game in which they did not participate. Despite the intentional programing of additional simulated play for bench players, they did not accumulate weekly loads similar to starting players. Overall, the weekly *integrated load* of bench players was only about 65% of the starters load (1,699 vs. 2,664 AU, respectively). These findings are unique, as most studies investigating external training load in basketball only include one type of playing group, such as starters (Bishop and Wright, 2006; Moreira et al., 2010), or only players that played the majority of the game minutes (Delextrat et al., 2012; Scanlan et al., 2015; Doeven et al., 2017; Puente et al., 2017; Sanders et al., 2018; Staunton et al., 2018; Vazquez-Guerrero et al., 2018, 2019; Alonso et al., 2020; Ransdell et al., 2020; Fox et al., 2020a). The present results highlight the importance of quantifying non-game activities to physically prepare all members of a basketball team.

Another novel finding of the present study was that players with 3- to 5-year experience spent the most time on court (~388 min/week) and had the highest weekly *integrated loads*. The total weekly durations for players in both the 1–2 and 3–5 year groups were moderately higher (*d* = 0.61 and *d* = 0.78, respectively) compared to players that had 10+ years’ experience. In the current study, players spent an average of ~340 min on court each week, which is similar to the ~368 min/week reported in semi-professional basketball players competing in three games per week (Fox et al., 2020b). Increased training load has previously been reported during weeks where 3 games were played, in both semi-professional (Fox et al., 2020b) and European professional (Salazar et al., 2020b) basketball. In this study, only 3/26 regular season weeks involved less than 3 games, meaning that “high game load” weeks in other leagues is normal practice in the NBA. While these differences are important to consider, the absolute volume of training undertaken by these players does not exceed 7 h per week on-court, even in the highest load periods. Therefore, it is likely that periodization of loading and recovery is more important than the absolute training volume. Determining “optimal” training prescription requires the context of other information (e.g., player responsiveness measures and basketball performance outcomes) and ideal periodization is likely different for each individual player (Salazar et al., 2020b).

Identifying differences between training load characteristics based on experience may inform approaches training management when transitioning through developmental pathways (e.g., high school and college) to the professional level better understand the training dose-response relationship over time, which could help plan future training programs and recovery strategies for high-value players. While the results are taken from one small cohort, it is the first study to compare external training load differences based on years of playing experience in NBA players, which we believe is important to consider when developing appropriate individualized training prescription. Previous research in Australian football, comparing external load based on years of experience, found that the most experienced group (7+ years) had the lowest in-season load (Rogalski et al., 2013). These authors suggested that age-related injury risk and resultant risk mitigation strategies could cause these differences (Rogalski et al., 2013). While the differences may be due to chance, our findings were similar in that players with less experience (i.e., ≤5 years in the NBA) had visually higher load than more experienced players, which may be due to an increased emphasis on player development during the early career phase, for these younger players. While development pathways will always be specific to each player, an understanding of physical demands through high school, college, and professional careers, combined with other contextual and individual factors (e.g., anticipated playing rotation) may help better plan training load for individual players beginning and throughout their NBA career.

To evaluate seasonal differences in *integrated load* and duration based on position, we dichotomized players to frontcourt or backcourt groups. While up to five traditional positions exist in basketball (point guard, shooting guard, small forward, power forward, center), previous research (Ribeiro et al., 2015; Vazquez-Guerrero et al., 2018; Reina Román et al., 2019) using such analyses with low samples of players (e.g., single club studies) limits the generalizability of the findings. A further complication of using a five-position classification is that players often play multiple positions during a season or within a single game. These fluid roles are becoming increasingly common in the NBA, where traditional positional classifications have evolved due to tactical changes including “small-ball” line ups (i.e., a line up not including a center) and “stretch 4’s” [i.e., a power forward (also referred to as the “4” position) with non-traditional offensive tactics] (Seidl et al., 2018). The present study showed no significant differences for *integrated load* or duration based on playing position. Previous research investigating external load in basketball by position has concluded that acceleration, deceleration, change of direction, and intensity demands varied based on position (i.e., centers, guards, and forwards; Svilar et al., 2018; Salazar et al., 2020a). However, the absence of differences between positions in the present study suggests that positional categorizations may be less important when evaluating global measures of external load (e.g., player load) and developing training plans throughout an NBA season. This is in line with the NBA moving away from traditional position roles and incorporating tactics, such as “small-ball” (Seidl et al., 2018). Although not evaluated in this study, it may still be important to consider individual roles, which are somewhat related to position, when evaluating very specific physical demands (i.e., contact and discrete movements) along with tactical requirements. This could lend insight to the difference in physical demands if players have more of an offensive or defensive role on the team, or provide information on how physical demands may change based on opponent or game strategies.

## Limitations

While this study advances current understanding of the physical demands experienced throughout an entire season, there are challenges in consolidating profiles of physical load in the NBA (McLean et al., 2018). One clear challenge is the investment from the players, highlighted in the current study where 4 players regularly chose not to wear a microsensor during non-game court activity. This contributed to the small number of participants for comparisons between groups, which reduces the statistical power of our analyses. The comparisons we made resulted in some players being included in the same groups across multiple categories (e.g., some starters were also frontcourt players), and the availability of players during practices and games, or lack thereof, could impact results. Overall, the low participant numbers (i.e., one team over one season) and missing data (i.e., no wearable data from 4/14 players) limits the generalizability of recommendations from the current findings.

To overcome these limitations in the NBA, it is important to create collaborative environments around player monitoring, which requires alignment from all stakeholders, including players, team staff, league officials and player unions. However, even with the most collaborative approaches, currently available technologies/systems are likely too cumbersome to apply during all on-court activity. One example of this is pre-game court work, in which players complete short (~15 min), predominantly individual, sessions before each game. The short time frame and technical focus of pre-game work means that collecting wearable data is highly impractical, but these short blocks of work represent a significant training load over an 82-game season, which may be important. As a result, we used *integrated load* estimates for 18.8% of the time spent on court (primarily from pre-game work), which could skew the results. However, we are confident that our estimates were reflective of the actual training load demands and, therefore, more valuable than excluding that training load altogether. While estimated and missing data are not ideal in research settings, missing data are often underreported in high-performance sport practice and research and not unique to basketball. In the NBA specifically, there are concerns from the players about the privacy and ownership of data generated from wearable technology that deters them from participating in team or league initiatives (Zillgitt, 2020). Through openly acknowledging and discussing these limitations we can move closer toward developing better solutions for player support.

Another challenge presented in this work is the need to integrate data from two systems that measure load differently. While we present one solution to integrate load data, this is far less desirable than a one system approach. A significant investment is required to understand the relationship between these systems, a luxury that may not be available to all practitioners facing similar challenges. Additionally, the approach and load measures used in the present study lack gold standard validity and present many logistical and data processing issues. Despite these limitations, the method we present for quantifying load does enable consistent, season-long information regarding the physical demands in the NBA. We again highlight that using only one data stream (e.g., publicly available game data) is insufficient for describing the demands that NBA players experience throughout a season.

Despite these limitations, the present findings provide novel information on physical demands and some of the associated contextual factors of the NBA, which improve current understanding and provide a platform for future work to build upon. Quantifying the individual physical demands is vital for enhancing player management and care in the NBA, where players have diverse training and playing backgrounds.

## Practical Applications

The present study provides novel information regarding practice and game load demands in the NBA. By integrating the duration and load demands of both practices and games across an entire NBA season, we highlight several factors that can impact training and recovery planning in NBA basketball. First, a significant portion of time and load accumulated in non-game activities has implications for player load management and periodization of court work throughout an NBA season. The findings related to duration and load demands across player categories emphasize the need for practitioners to develop integrated and consolidated monitoring systems to best inform individualized training prescription and optimize preparation of NBA players. Additionally, this study highlights some limitations to conducting applied research in a high-performance environment (e.g., low participant numbers, missing data, and data from multiple sources) which are often underreported. Reporting these limitations in the NBA is novel and represents one of the major contributions of this work, as it provides additional information and context for stakeholders seeking to improve current systems; we strongly encourage other researchers to acknowledge such limitations in their work. Future studies utilizing multi-center or league-wide approaches would strengthen the depth and breadth of understanding around player and training characteristics so that more generalizable recommendations can be made. Collaborative approaches are imperative within high-performance environments, in order to develop integrated player monitoring solutions and continue to educate stakeholders about the value of training load monitoring, in order to support best practices for player preparation.

## Conclusion

This is the first study to evaluate the holistic load demands of NBA players across an entire season. This study described the time NBA players spent in basketball-specific activity and highlights that a significant portion of time and load is accumulated in non-game activities. The present results identified that duration was significantly higher for players with 3–5 years of NBA experienced compared to players with <3 years or ≥ 6 years. *Integrated load* was significantly higher for starters compared to bench players, while total load did not appear to be significantly impacted based on playing position.

## Data Availability Statement

The raw data supporting the conclusions of this article will be made available by the authors, in a format whereby individual participants are not identifiable, and without undue reservation.

## Ethics Statement

The study was approved by the Human Research Ethics Committee of the University of Technology Sydney (UTS; HREC # ETH18-2658). Written informed consent from participants was not required for this study. Consent to analyze and publish this data was granted by the NBA and the NBA Players Association as per the guidelines and requirements for “NBA related health research” outlined by the NBA Collective Bargaining Agreement (CBA; NBA.com, 2017).

## Author Contributions

JR, BM, DS, and AC contributed to conception and design of the study. JR and BM collected and organized the data. SS performed the statistical analysis. JR wrote the first draft of the manuscript. All authors contributed to manuscript revision, read, and approved the submitted version.

## Conflict of Interest

BM, JR and DS involved in this work are NBA affiliated practitioners/researchers. As such, the study design and methods have been required to comply with the NBA Health-Related Research policy. This work has been reviewed by the NBA, NBA Physicians Association, NBA Players Association. As part of this process, this manuscript was made available for comment from the NBA and NBA Research Committee prior to publication (these contributors are not listed as authors). The authors declare an absence of any other commercial or financial relationships that could be construed as a potential conflict of interest.

## Publisher’s Note

All claims expressed in this article are solely those of the authors and do not necessarily represent those of their affiliated organizations, or those of the publisher, the editors and the reviewers. Any product that may be evaluated in this article, or claim that may be made by its manufacturer, is not guaranteed or endorsed by the publisher.
